# MRI findings of the brain in human African trypanosomiasis: a case series and review of the literature

**DOI:** 10.1259/bjrcr.20180039

**Published:** 2018-06-18

**Authors:** Nikhil K Patel, Arthur Clegg, Michael Brown, Harpreet Hyare

**Affiliations:** 1 Department of Radiology, King's College Hospital, London, UK; 2 Department of Infectious Diseases, University College Hospital, London, UK; 3 Department of Infectious and Tropical Diseases, Hospital for Tropical Diseases, London, UK; 4 Department of Radiology, University College Hospital, London, UK

## Abstract

Human African Trypanosomiasis (HAT) is a neglected tropical disease that affected 3797 people worldwide in 2014. Without treatment mortality approaches 100%. Due to its low incidence and non-specific clinical features, diagnosis can be challenging and the role of MRI in diagnosis of HAT has not been evaluated outside of case reports. We carried out a retrospective, institutional review of three patients presenting with neurological stage (Stage 2) HAT presenting to the Hospital of Tropical Diseases, London between 2004 and 2016. MRI brain was performed in both the acute and follow-up stages of their infection. In addition to confirming that the most common MRI abnormality is *T*_2_ weighted fluid-attenuated inversion recovery (T2W FLAIR) high signal intensity in the supratentorial white matter, this series has identified radiological findings not previously reported in the literature. In the acute stages, restricted diffusion can be seen in the internal capsules and splenium of the corpus callosum and microhaemorrhages not related to melarsoprol have been identified. Furthermore, the signal abnormality appears to be largely reversible upon treatment with regression associated with mild atrophy demonstrated on follow-up MRI post-treatment. We conclude that although direct microscopy remains the mainstay of diagnosis with serological and polymerase chain reaction (PCR) testing providing useful adjuncts, MRI brain can be helpful in assessing neurological involvement and may provide important prognostic information post-treatment.

## INTRODUCTION

Human African Trypanosomiasis (HAT) is a protozoan infection caused by Trypanosoma brucei. Two subspecies infect humans: T.b. gambiense which has a reservoir in humans and is found in West and Central Africa, and the zoonotic T.b. rhodesiense found in East Africa. Both are vector borne and spread via the Tsetse fly (Glossina sp). Infection is divided into two stages. The initial haemolymphatic stage (Stage 1) is characterised by fever, lymphadenopathy, hepatosplenomegaly and anaemia - this progresses to the second neurological stage (Stage 2) which is characterised by a broad range of neurological symptoms including distortion of the sleep-wake cycle. Without treatment, mortality approaches 100%.^1^ T.b. gambiense [West African trypanosomiasis (WAT)] progresses from inoculation to death over a period of months to years, with T.b. rhodesiense [East African trypanosomiasis (EAT)] progressing over weeks to months. Accurate classification into WAT, EAT and Stage 1 and 2 disease is essential due to differences in diagnosis, treatment and outcomes.

In 1995 the World Health Organization (WHO) estimated that there were 300,000 new cases of HAT in Africa each year.^2^ Eradication programs have seen this number fall sharply with 3679 cases of WAT^3^ and 118 cases of EAT reported in 2014,^4^ however, 55.1 million people are still at risk of the disease.^3^ Cases diagnosed outside of Africa number <50 for both WAT and EAT each year.^5^

Due to its low incidence and non-specific clinical features, diagnosis of HAT can be challenging. Definitive diagnosis of HAT relies on isolation of the trypanosomes from blood, lymph node aspirate or cerebrospinal fluid (CSF) in the case of Stage 2 disease, though serological and PCR diagnostic methods are of increasing value. Due to the paucity of MRI in endemic countries, its role in diagnosis of HAT has not been evaluated outside of case reports. Here we report a case series of three patients (two WAT and one EAT) who presented to the Hospital of Tropical Diseases, London between 2004 and 2016 who underwent MRI imaging in both the acute and follow-up stages of their infection.

Clinical aspects of these cases have already been reported in the literature by our institution.^6–8^

## CASE REVIEW

### Case 1—West African Trypanosomiasis

A 58-year-old Nigerian national living in the Delta state presented to a district general hospital in the UK in August 2016 with a history of fever, confusion and drowsiness. Her illness had started in January 2016 with lethargy and tremors and for the previous 2 months this had progressed to unsteadiness on walking, 18 kg weight loss and increasing malaise. On arrival she had no focal neurology, signs of meningism or lymphadenopathy but was found to have poor co-ordination with myoclonic jerks and bradyphrenia.

Her admission bloods showed a white cell count (WCC) of 5.4 × 10^9^ l^–1^ with a monocytosis, microcytic anaemia, thrombocytopenia and a C-reactive protein (CRP) of 13 mg l^−1^. Her total protein was elevated at 84 g l^−1^ with a globulin of 47 g l^−1^. Malaria film was negative. HIV and syphilis serology were negative. CT head was unremarkable.

Admission CSF showed a WCC of 331 (99% mononuclear), a protein of 0.82 g l^−1^ (0.23–0.38) and a glucose of 3.1 mmol l^−1^ (>50% serum level). Herpes virus, enterovirus and JC virus PCR of the CSF was negative. CSF genXpert and mycobacterial culture were negative.

She was initiated on antiviral and antituberculous treatment.

Due to her deteriorating condition serum was sent for T.b. gambiense Indirect Fluorescent Antibody Test which was positive at 1:400 and she was transferred to the Hospital for Tropical Diseases.

Repeat lumbar puncture (LP) on admission showed a WCC of 1140 (90% mononuclear), a protein of 1.14 g l^−1^ and a glucose of 2.3 mmol l^−1^ (serum 5.5). CSF T.b. gambiense IFAT was positive at 1:4. No trypanosomes were detected in peripheral blood or CSF. PCR of CSF and serum was positive with primers specific for T.b. gambiense Type 1.

**Figure 1.**
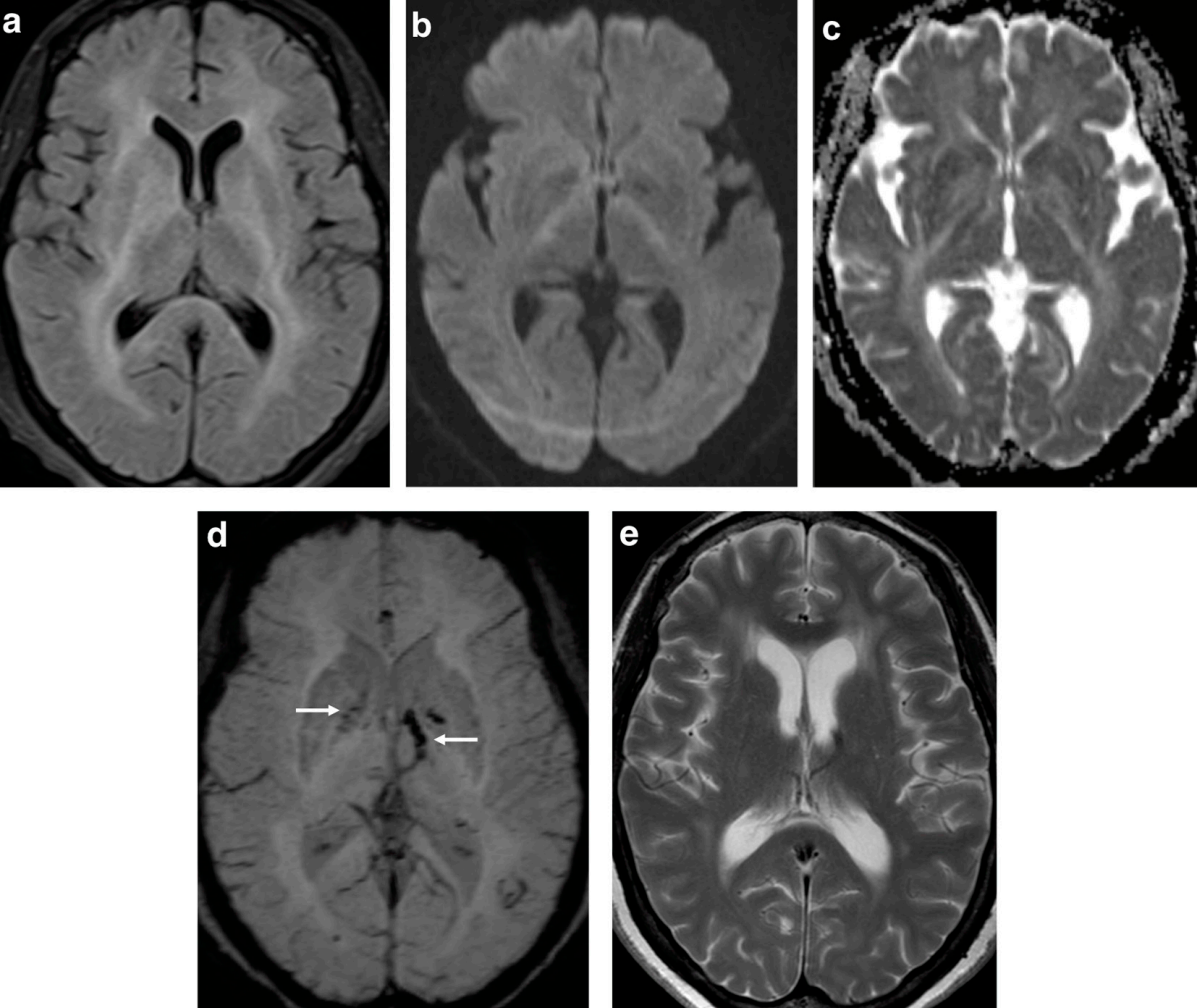
58-year-old female with West African Trypanosomiasis. (a) Axial T2W FLAIR demonstrates bilateral hyperintense signal involving the deep white matter, basal ganglia, internal and external capsule as well as the splenium of the corpus callosum. (b)DWI showing subtle high signal intensity in the posterior limbs of the internal capsules with corresponding low signal on the ADC map consistent with restricted diffusion (c). (d) SWI demonstrating blooming artefact in the globus pallidus bilaterally and left internal capsule suggestive of microhaemorrhages (arrows). (e) Axial *T*_2_ performed 4 months later shows interim development of ventriculomegaly and some residual deep white matter signal abnormality. ADC, apparent diffusion coefficient; DWI, diffusion-weighted imaging; SWI, susceptibility-weighted imaging; T2W FLAIR, *T*_2_ weighted fluid-attenuated inversion recovery.

MRI was performed with a *T*_2_ weighted fluid-attenuated inversion recovery (T2W FLAIR) sequence demonstrating diffuse, symmetrical high signal intensity of the deep white matter involving the basal ganglia and splenium of the corpus callosum (Figure 1a). Diffusion-weighted imaging (Figure 1b) and apparent diffusion coefficient (ADC) map (Figure 1c) highlighted subtle restricted diffusion confined to the posterior limbs of the internal capsules with susceptibility-weighted imaging (SWI) demonstrating microhaemorrhages involving the globus pallidus, left internal capsule and thalamus (Figure 1d).

She was treated for Stage 2 WAT with nifurtimox/eflornithine combination therapy as per the WHO guidelines.

She made a good clinical recovery with resolution of her tremor, fever, myoclonus and confusion. She remained well at her 4-month review with repeat imaging demonstrating resolution of the restricted diffusion, improvement in the deep white and grey matter signal abnormality and mild supratentorial atrophy (Figure 1e)

### Case 2—West African Trypanosomiasis

A 62-year-old male born in Sierra Leone presented to a UK hospital in January 2012 with a 3-month history of personality change, somnolence, shuffling gait and fatigue. Examination revealed right side lateral gaze palsy with rigidity and bradykinesia. A few days into his admission he developed fevers with progressive somnolence.

Admission CSF showed a WCC of 250 (100% mononuclear) and a protein of 0.57 g l^−1^ with a normal CSF/serum glucose ratio. Herpes virus PCR was negative.

HIV and syphilis serology were negative. He was found to have high levels of voltage gated potassium channel-complex antibodies with moderate levels of N-methyl-D-aspartate antibodies. He was initially treated with i.v. acyclovir and ceftriaxone with no effect.

He suffered a generalised tonic-clonic seizure and unresponsiveness that required intubation and intensive therapy unit (ITU) admission. Microscopy of a bone marrow trephine revealed Trypomastigotes of T. brucei, which were subsequently seen in peripheral blood.

He was transferred to the Hospital for Tropical Diseases and commenced on nifurtimox/eflornithine combination therapy as per the WHO guidelines for a Stage 2 WAT. Repeat CSF showed a positive T.b. gambiense IFAT at 1:32 and a serum IFAT was positive at 1:3200.

**Figure 2.**
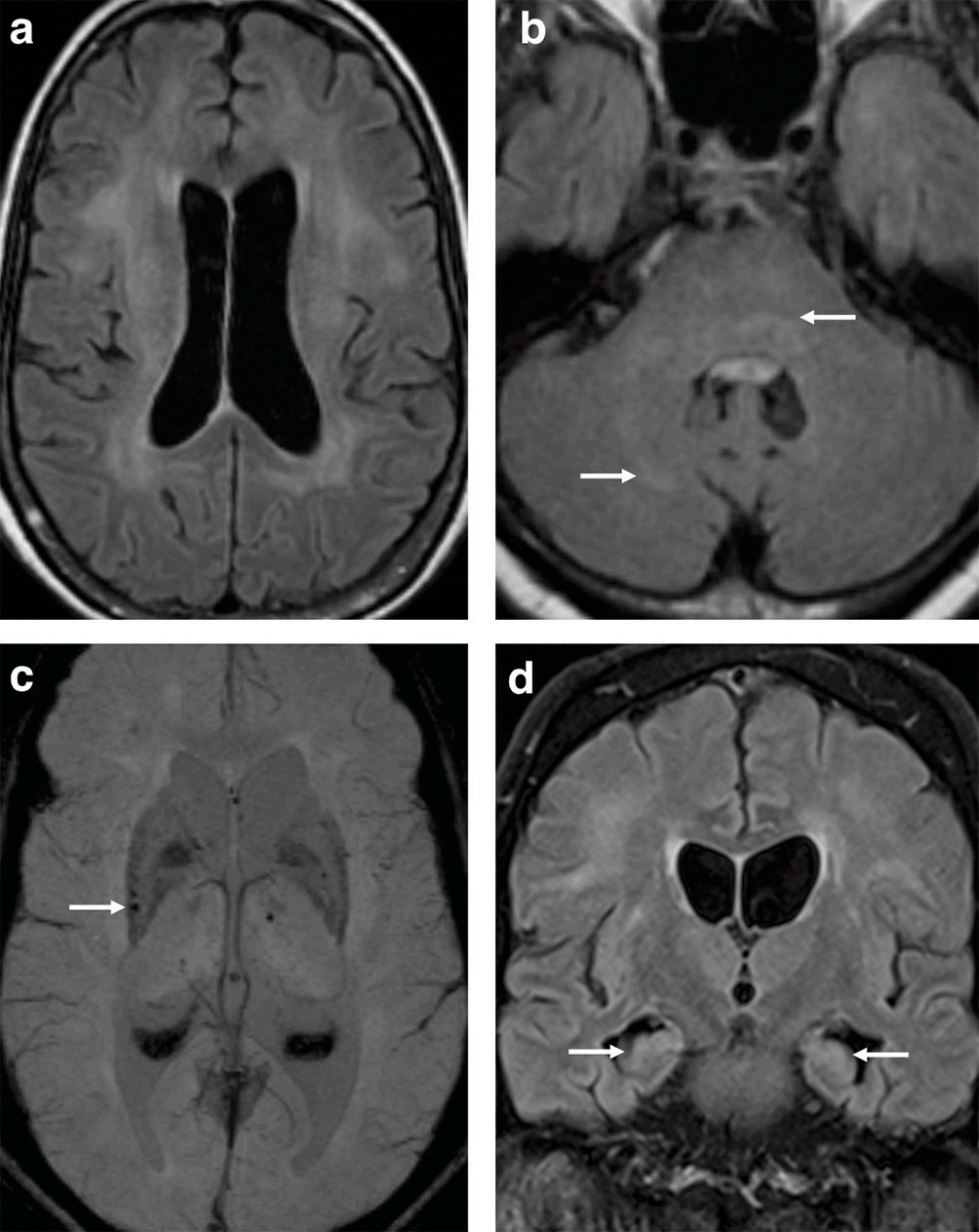
62-year-old male with West African Trypanosomiasis. (a,b) Axial T2W FLAIR demonstrating ventriculomegaly with bilateral deep white matter hyperintense signal extending from the corona radiata into the posterior limbs of the internal capsules (not shown) as well as the mid brain, pons and right cerebellum (arrows). MRI performed 3 months later shows new susceptibility artefact on SWI in the right posterior putamen (arrow) (c). (d) Coronal T2W FLAIR demonstrates some improvement in bilateral white matter changes and stable ventricular size with hyperintense signal in the hippocampi (arrows). SWI, susceptibility-weighted imaging; T2W FLAIR, *T*_2_ weighted fluid-attenuated inversion recovery.

T2W FLAIR MRI performed on transfer showed bilateral supratentorial deep white matter high signal intensity (Figure 2a) extending to involve the cerebellum and brain stem (Figure 2b) as well as the mesial temporal lobe structures. The ventricles appeared prominent for his age.

4 days into treatment he was extubated with reversal of his coma and he was transferred to the ward. On day 9 of treatment he became drowsy. CSF showed no evidence of trypanosomes. Given his previously high VGKC-complex antibodies he was treated with 9 cycles of plasma exchange with good effect.

At 1-month post-discharge his mobility and cognition had improved and his extrapyramidal symptoms had resolved. An MRI performed 3 months following the initial scan showed new blooming artefact on SWI in the pons, putamen and thalamus (Figure 2c) with improvement in the deep white matter signal abnormality and residual high signal in the hippocampi (Figure 2d).

### Case 3—East African Trypanosomiasis

A 38-year-old British male presented to a South African hospital in August 2004 after 2 years of travelling in South Africa, Malawi, Mozambique and Namibia. He presented with a 4-month history of progressive fatigue, fever, headache and sleeplessness.

On arrival he was found to be febrile with mild hepatomegaly and lymphadenopathy. Bloods showed raised inflammatory markers with a CRP of 54 mg l^−1^ and an of 120. CSF showed a raised protein at 1.2 g l^−1^ with a glucose level of 2.1 mmol l^−1^, the WCC was 82 (100% mononuclear). CT head was unremarkable.

An HIV test was negative. A blood film was positive for trypanosomes and a diagnosis of Stage 2 EAT was made. He was treated with suramin and melarsoprol with prednisolone cover. He made a full recovery and 2 weeks post-treatment his CSF had normalised.

In June 2005 he reported a 2-month history of headache, night sweats, somnolence and fever. Repeat LP showed a WCC of 58 (100% mononuclear), a protein of 0.79 g l^−1^ and a glucose of 2.9 mmol l^−1^. Blood and CSF showed no trypanosomes. He was treated as a relapsed trypanosomiasis and was commenced on a 2-week course of eflornithine from which he made a rapid recovery. His CSF WCC count fell to normal levels by August 2005.

In December 2005 he presented with a 2-week history of headache, fever, vertigo, diplopia and somnolence. He had a left sixth nerve palsy on examination. LP showed trypanosomes in his CSF with a WCC of 125 (100% mononuclear), a protein of 0.8 g l^−1^ and a glucose of 3.1 mmol l^−1^. His blood was positive for trypanosomes. He was treated with a repeat course of suramin and melarsoprol with prednisolone cover for relapsed Stage 2 EAT.

**Figure 3.**
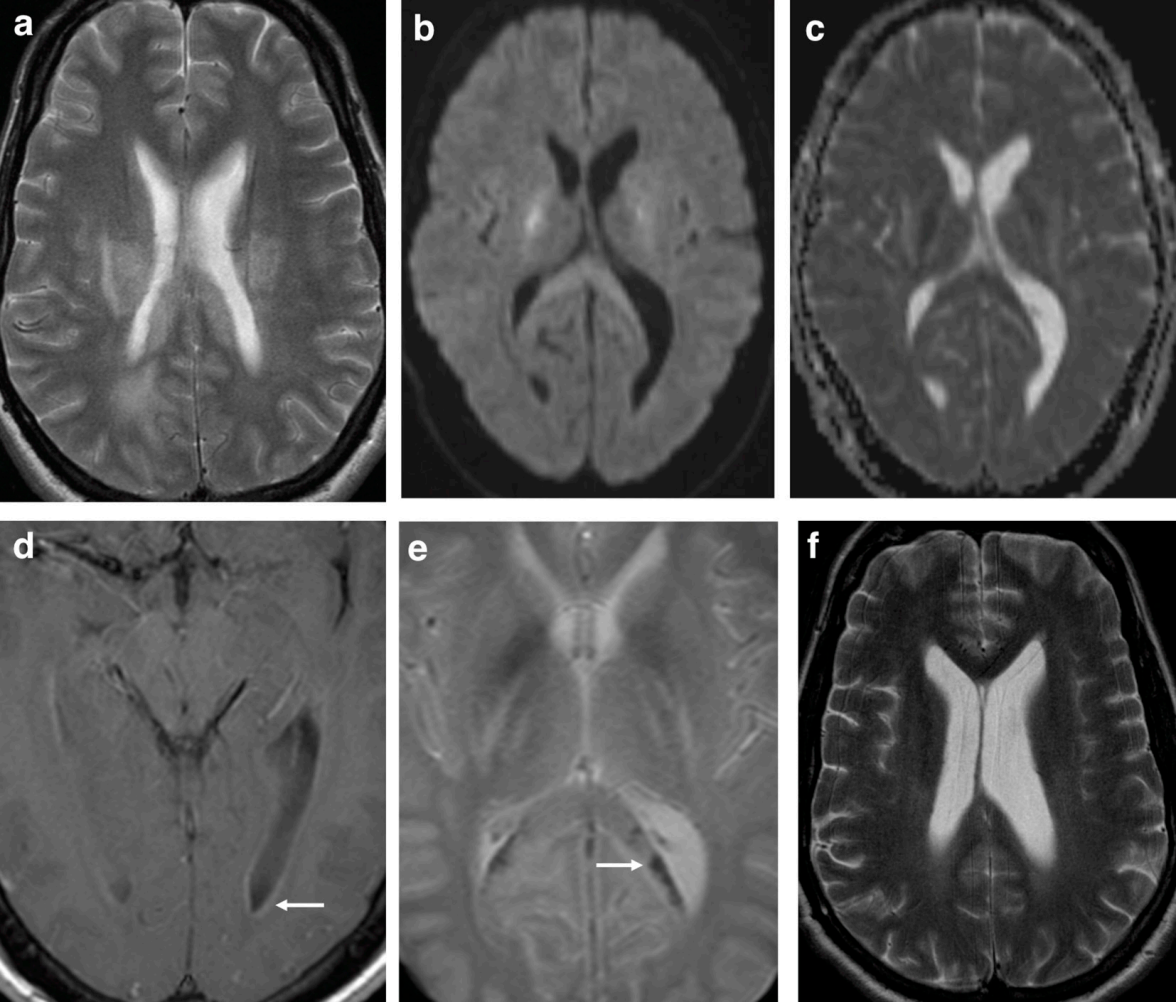
38-year-old male with East African Trypanosomiasis. (a) Axial *T*_2_ showing bilateral hyperintense signal of the deep white matter. (b) Axial DWI and ADC map (c) demonstrates true restricted diffusion in the splenium of the corpus callosum and posterior limbs of the internal capsule. (d) Gadolinium enhanced axial MRI showing subtle ependymal enhancement of the posterior horns of the lateral ventricles (arrow). (e) Axial *T*_2_* demonstrating blooming artefact in keeping with microhaemorrhages in the splenium of the corpus callosum (arrow). (f) T2 axial MRI performed 6 months later showing improvement in hyperintense deep white matter signal with interim ventricular enlargement. ADC, apparent diffusion coefficient; DWI, diffusion-weighted imaging; *T*_2_*, *T*_*2*_-star.

On day 16 of his treatment he became confused and suffered a generalised tonic-clonic seizure. LP showed an opening pressure of 34cmH2O with no cells and a protein of 1.06 g l^−1^. An MRI at this time demonstrated T2W hyperintense signal in the supratentorial white matter extending into the posterior limbs of the internal capsules and splenium of the corpus callosum (Figure 3a), both of which showed corresponding restricted diffusion (Figure 3b,c). Post-contrast sequences revealed mild ependymal enhancement of the occipital horns of the lateral ventricles with no parenchymal enhancement (Figure 3d). Focal signal loss on *T*_2_-star (*T*_2_*) imaging was also present in the splenium of the corpus callosum and basal ganglia (Figure 3e) in keeping with microhaemorrhages and he was transferred to ITU where he was sedated and intubated.

Follow-up imaging 6 months later showed residual but reduced T2W FLAIR signal hyperintensity in the deep white matter, resolution of the restricted diffusion abnormalities and mild supratentorial atrophy when compared to his admission MRI (Figure 3f).

## DISCUSSION

The most consistent brain MRI finding in the three cases presented is diffuse symmetrical deep white matter T2W FLAIR hyperintensity with less striking involvement of the splenium of the corpus callosum, basal ganglia, mid brain and pons. Follow-up imaging post-treatment demonstrated marked regression of the signal abnormality associated with mild atrophy in two of the three cases over a 4 to 6-month period.

The imaging findings in HAT have been described as far back as 1989— a literature review identified 13 publications (12 unique patients) with specific reference to MRI findings of the brain. Seven patients were diagnosed with WAT, four with EAT and one unknown (Table 1).

**Table 1.** Published MRI findings in HAT

**Author and year of publication**	**MRI findings**	**Follow-up imaging**
Spinazzola et al 1989^9^ (WAT)	↑SI frontal and temporal white matter	None
Serrano-Gonzalez et 1996^10^ (WAT)	Bilateral white matter ↑SI extending into basal nuclei and brainstem	Resolution of signal abnormalities
Sabbah et al 1997^11^ (EAT)	↑SI in the internal capsules, cerebellum and splenium of corpus callosum with meningeal thickening	None
Bedat-Millet et al 2000^12^ (WAT)	Bilateral white matter and cerebellar ↑SI	None
Sahlas et al 2002 and Gill et al 2003^13, 14^ (WAT)	↑SI in the basal ganglia, internal and external capsules with little contrast enhancement	1-year follow up: residual ↑SI and ventricular enlargement
Braakman et al 2006^15^ (EAT)	Bilateral ↑SI of deep white and grey matter	None
Kumar et al 2006^16^ (EAT)	Bilateral ↑SI in subcortical white matter, cerebellum, brainstem and cervical spine	None
Checkley et al 2007^8^ (EAT)	↑SI in internal capsule and corpus callosum with microhaemorrhages (PTRE)	None
Kager et al2009^17^ (WAT)	Bilateral ↑SI of deep white matter, basal ganglia and mesencephalon	4 years follow up: remnant ↑SI and ventricular enlargement
Gillmore et al 2009^18^ (Unknown)	Diffuse ↑SI in the deep white matter with less striking changes in the deep grey matter and brainstem	None
Liu et al 2010^19^ (WAT)	↑SI of the subcortical white matter, midbrain and pons	None
Wengert et al 2014^20^ (WAT)	↑SI in hemispheric deep white matter, deep grey matter and brain stem.	2 years follow up: subtle ventricular enlargement and resolution of ↑SI

HAT, human African trypanosomiasis; WAT, West African trypanosomiasis; EAT, East African trypanosomiasis; ↑SI, high signal intensity ; PTRE, post-treatment reactive encephalopathy.

All published cases of MRI in HAT have symmetrical high T2W FLAIR signal abnormalities of the deep white matter tracts, with involvement of the basal nuclei seen less frequently in only 6 of the 12 cases.^10, 14,15,17,18,20^ Signal abnormalities of the cerebellum and brainstem occurred in just over half of published reports (seven cases^10,12,16–20^ with ventricular enlargement and varying degrees of improvement in signal abnormalities in the four cases that had follow-up imaging.^10, 14,17,20^ The signal abnormality may be the result of perivascular infiltration with surrounding areas of demyelination and axonal damage as seen on autopsy.^15^ Atrophy of the brain – thought not to be described in autopsy reports as the patients usually had active disease – is only apparent as a late effect with MRI demonstrating these changes particularly well.^17^

Microhaemorrhage on MRI, seen as blooming artefact on SWI and T2*W imaging, is seen in the three cases presented in this series. In the first case, microhaemorrhages were seen in the globus pallidus, left internal capsule and left periventricular deep white matter while the second demonstrated smaller foci in the pons, thalamus and putamen. Cerebral microhaemorrhage and axonal injury have been described in the chronic stages of the disease on histopathology^21^ and these findings have not been previously reported on MRI in HAT. At our institution, a haemorrhage sensitive sequence is part of our routine diagnostic MRI brain protocol and it may be that this radiological finding may have been previously under-reported in this disease. Indeed, the third case of microhaemorrhage was previously attributed to melasoprol-induced post-treatment reactive encephalopathy (PTRE)^8^ corresponding to pathology,^22^ but in fact may be a radiological feature of this disease.

Restricted diffusion in the posterior limbs of the internal capsules was seen in two of our cases with restricted diffusion in the splenium of the corpus callosum seen in Case 3. The presence of restricted diffusion in the posterior limbs of the internal capsules and splenium of the corpus callosum has not been reported previously in cases of HAT. Known as the "boomerang’ sign, this transient lesion in the splenium of the corpus callosum has been described in a wide range of clinical conditions including epilepsy, encephalitis (caused by a variety of organisms including tick borne and H1N1), metabolic (*e.g.* hypoglycaemia and renal failure), demyelination and vascular causes to name a few.^23^ In HAT, the restricted diffusion could be a marker of acute neurological involvement, resolving on follow-up imaging post-treatment and likely to be due to the known perivascular infiltration seen in the acute stages.

Accurate and definitive diagnosis of HAT still requires identification of the parasite within blood, lymph node aspirate or fluid from a chancre under microscopy, although it is not uncommon to fail to identify trypanosomes in cases of T.b. gambiense. Serological testing in patients with a high index of suspicion is required in this situation. Although the MRI findings in HAT are largely non-specific, MRI may be helpful in staging the disease and providing prognostic information upon follow up. Analysis of CSF should be performed in order to confirm the MRI suspicion of central nervous system involvement and therefore select the most appropriate therapy.

This case series brings new MRI imaging findings not previously reported in HAT. In the acute stages, restricted diffusion can be seen in the internal capsules and splenium of the corpus callosum and microhaemorrhages not related to melarsoprol have been identified. In addition, the signal abnormality appears to be largely reversible upon treatment with regression associated with mild atrophy that can occur within a relatively short period of time ranging from 4 to 6 months.

## LEARNING POINTS

1. Although the MRI findings in HAT are non-specific, the presence of supratentorial deep white matter signal change in a patient with a relevant travel history should alert the clinician to a potential diagnosis of Stage 2 HAT infection.
2. Restricted diffusion may be a sign of acute infection that resolves upon treatment.
3. Direct microscopy remains the mainstay of HAT infection with serological and PCR testing providing useful adjuncts.
